# Genomic and small RNA sequencing of *Miscanthus × giganteus *shows the utility of sorghum as a reference genome sequence for Andropogoneae grasses

**DOI:** 10.1186/gb-2010-11-2-r12

**Published:** 2010-02-03

**Authors:** Kankshita Swaminathan, Magdy S Alabady, Kranthi Varala, Emanuele De Paoli, Isaac Ho, Dan S Rokhsar, Aru K Arumuganathan, Ray Ming, Pamela J Green, Blake C Meyers, Stephen P Moose, Matthew E Hudson

**Affiliations:** 1 Department of Crop Sciences, University of Illinois at Urbana-Champaign, Urbana, IL 61801, USA; 2 Energy Biosciences Institute and Institute for Genomic Biology, University of Illinois at Urbana-Champaign, Urbana, IL 61801, USA; 3 Department of Plant and Soil Sciences, Delaware Biotechnology Institute, University of Delaware, Newark, DE 19716, USA; 4 DOE Joint Genome Institute, Walnut Creek, CA 94598, USA; 5 Center for Integrative Genomics, University of California at Berkeley, Berkeley, CA 94720, USA; 6 Benaroya Research Institute at Virginia Mason, Seattle, WA 98101, USA; 7 Department of Plant Biology, University of Illinois at Urbana-Champaign, Urbana, IL 61801, USA; 8 College of Marine Studies, University of Delaware, Newark, DE 19711, USA

## Abstract

**Background:**

*Miscanthus × giganteus *(*Mxg*) is a perennial grass that produces superior biomass yields in temperate environments. The essentially uncharacterized triploid genome (3n = 57, x = 19) of *Mxg *is likely critical for the rapid growth of this vegetatively propagated interspecific hybrid.

**Results:**

A survey of the complex *Mxg *genome was conducted using 454 pyrosequencing of genomic DNA and Illumina sequencing-by-synthesis of small RNA. We found that the coding fraction of the *Mxg *genome has a high level of sequence identity to that of other grasses. Highly repetitive sequences representing the great majority of the *Mxg *genome were predicted using non-cognate assembly for *de novo *repeat detection. Twelve abundant families of repeat were observed, with those related to either transposons or centromeric repeats likely to comprise over 95% of the genome. Comparisons of abundant repeat sequences to a small RNA survey of three *Mxg *organs (leaf, rhizome, inflorescence) revealed that the majority of observed 24-nucleotide small RNAs are derived from these repetitive sequences. We show that high-copy-number repeats match more of the small RNA, even when the amount of the repeat sequence in the genome is accounted for.

**Conclusions:**

We show that major repeats are present within the triploid *Mxg *genome and are actively producing small RNAs. We also confirm the hypothesized origins of *Mxg*, and suggest that while the repeat content of *Mxg *differs from sorghum, the sorghum genome is likely to be of utility in the assembly of a gene-space sequence of *Mxg*.

## Abstract 2

Genomic data together with sequencing of tissue specific small RNA libraries reveals insights into the genome content, small RNA repertoire and evolutionary origins of the grass *Miscanthus × giganteus*.

## Background

Domesticated and wild grass species in the Andropogoneae tribe are important as sources of food, feed, fiber and fuel. Included within the Andropogoneae are major crops such as maize, *Sorghum bicolor *(sorghum), sugarcane (*Saccharum *spp.), the native North American prairie grass *Andropogon gerardii *(Big Bluestem) and species in the genus *Miscanthus*, which have recently emerged as leading candidate bioenergy crops. All species within the Andropogoneae perform C4 photosynthesis, which exhibits higher rates of carbon fixation. C4 is also associated with greater water- and nutrient-use efficiency compared to most plant species [1,2]. These physiological properties contribute to the very high biomass productivity observed from cultivated Andropogoneae crop species, which often exceeds 20 Mg/ha. Another characteristic of the Andropogoneae is a high frequency of polyploidy, with many large and complex genomes. Though typically native to tropical and subtropical climates, Andropogoneae crop species are adapted to a wide diversity of environments and vary in both their life cycle (annual versus perennial) and primary form of harvestable carbon (sugar, grain starch or cellulose).

As a result of their global economic importance as crops, their varied biology and diversity of genome structure, Andropogoneae species have been an important focus for plant genomics research. Early comparisons of genetic maps and local genome structure helped elucidate the high degree of synteny among grass genomes [3]. These and other studies have also provided insights into the contributions of transposon content, gene duplication, and conserved noncoding sequences to plant genome evolution [4,5]. Recently, whole-genome shotgun sequencing has produced a high coverage draft for one of the smallest known Andropogoneae genomes, sorghum [6]. In addition, a strategy to sequence the maize genome using assembled BAC contigs that are anchored to genetic and physical maps is now complete [7], and extensive EST resources exist for *Saccharum officinarum *[8].

The *Miscanthus *genus is composed of about 15 species of rhizomatous perennial grasses native to subtropical and tropical regions of Africa and southern Asia, with the range of at least four species (*M. sinensis*, *M. sacchariflorus*, *M. floridulus *and *M. × giganteus*) extending north into temperate eastern Asia [9]. Sequencing of nuclear ribosomal DNA and plastid intergenic spacers as well as amplified fragment length polymorphism genotyping has resolved some of the phylogenetic relationships among *Miscanthus *spp. and accessions [9-11]. Within the Andropogoneae, *Miscanthus *is most closely related to *Saccharum *spp. and interspecific hybrids of *Miscanthus *and *Saccharum *have been used in genetic improvement of sugarcane [12].

*Miscanthus × giganteus *[13,14] (hereafter referred to as *Mxg*) is widely distributed as an ornamental and has been evaluated for more than two decades as a biomass crop in both Europe and the United States [15,16]. Because of these attributes, *Mxg *has been the most extensively studied member of the *Miscanthus *genus. *Mxg *is a sterile triploid (3n = 57, x = 19) that likely originated from the hybridization of *M. sinensis *and *M. sacchariflorus *[11,17,18]. In addition to the potential for increased vigor because of stabilized hybridity and polyploidy, *Mxg *is unique among C4 grasses in performing C4 photosynthesis at temperatures as low as 5°C [19,20], which likely contributes to a maximal 2% conversion rate of solar energy into harvestable biomass among established canopies [16]. This phenotype has been interpreted as a competitive advantage since it allows productive photosynthesis to begin earlier in spring in temperate climates. The cold-tolerant C4 photosynthesis phenotype is correlated with increased accumulation of the key C4 photosynthetic enzyme pyruvate orthophosphate dikinase under chilling conditions [21,22].

Future studies of *Mxg *and other *Miscanthus *species, as well as genetic improvement of these species as biomass crops, will be enhanced by knowledge of their genome content and organization. The *Mxg *genome also offers the possibility to gain information about examples of both the *M. sinensis *and *M. sacchariflorus *genomes, and their interactions after hybridization. As a consequence of both its interesting biological properties and potential as a biomass crop, *Mxg *is a desirable target for whole-genome sequencing. Although the large size, complexity, and often polyploid nature of Andropogoneae genomes makes whole-genome shotgun or map-based strategies cost-prohibitive, short-read deep sequencing technologies enable rapid cost-effective approaches to survey genome content and organization [23]. Such an approach is greatly enhanced by comparisons to a closely related reference genome sequence [24,25] and represents an efficient first step toward characterizing complex eukaryotic genomes. We reasoned that the recently completed whole-genome shotgun genome sequence for sorghum [6] would likely provide an appropriate initial reference sequence for comparative analysis with the *Mxg *genome, both for the characterization of conserved sequences as well as recently diverged repeats and gene content found in *Mxg *but not sorghum. The comparison of repeat composition between these two genomes has not yet been performed, nor is it known how similar genic sequences are likely to be between *Mxg *and available reference genomes. We thus evaluated this strategy by comparing a survey of the *Mxg *genome obtained by 454 Genome sequencer FLX system (GS-FLX) pyrosequencing to the genome sequences of sorghum, maize and rice. We also compared the repeat sequences identified using the survey results with profiles of small RNAs (sRNAs) obtained via Illumina sequencing-by-synthesis as it has been previously reported that repetitive regions of plant genomes contribute a substantial portion of the total sRNA transcriptome [26].

## Results

### Genome size estimation for *Miscanthus *species

We used flow cytometry to estimate nuclear DNA contents from the exact clone used for this study, a single accession of *Mxg *named 'UIUC', which was originally established as an ornamental variety at the University of Illinois at Urbana-Champaign, but is also the source of material used in recent agronomic trials [16]. We obtained values ranging from 7.60 to 7.95 pg among four independent tissues samples for the UIUC accession of *Mxg *(Additional file 1). These values are closely consistent with those previously published for *Mxg *[27]. Cells in G0/G1 were identified using flow cytometry and comparison to size standards (Additional file 2) and are consistent with previous measurements [27]. Using a value of 980 Mbp per pg DNA [28], and assuming *Mxg *harbors three genomes of similar size, we estimate that 3C = 7.5 Gbp, and thus the three haploid genomes of *Mxg *are approximately 2.5 Gbp.

### Survey sequencing of *Mxg*

We performed a whole-genome survey of the *Mxg *genome using the 454 sequencing platform as described previously for soybean [23]. Genomic DNA was isolated from nuclei purified from *Mxg *leaves to minimize possible contamination from organellar or microbial DNA. A total of 84 Mbp in 366,448 reads with an average size of 229 bp were produced from one run using the 454 LR70 FLX technology. A maximum value for chloroplast contamination (reads with a BLASTN hit to the sorghum chloroplast genome at e < 10^-6^) was 0.24% (901 reads). A maximum value for mitochondrial contamination (reads with a BLASTN hit to the sorghum mitochondrial genome at e < 10^-6^) was 0.21% (817 reads). These are maximum values because the nuclear genome may contain sequences identical or near-identical to the chloroplast and mitochondrial genomes; the 0.24% figure is similar to the proportion of the *Arabidopsis *nuclear genome composed of integrated chloroplast sequences [29]. The average GC content of the sequence was 44%.

Hodkinson [11] suggested that the triploid genome of *Mxg *is most likely derived from the cross of a diploid *M. sinensis *or *M. sacchariflorus *with a fertile *M. sinensis *× *M. sacchariflorus *allotetraploid, each of which are known to exist in Japan, where *Mxg *is thought to have originated. Observed patterns of meiotic chromosome pairing [17] support the view that *Mxg *harbors two genomes of high homology and a third with greater divergence. The component genomes of *Mxg *share sufficiently high similarity to prevent specific detection by genome *in situ *hybridization [11], but the two genomes can be distinguished by sequence variation in the internal transcribed spacer of 18-28S rDNA genes (rDNA is DNA encoding the tandem array of rRNA genes). *Mxg *is sterile due to frequent chromosomal mispairing during meiosis [17]. For the purpose of determining coverage, we propose that *Mxg *is best treated as a heterozygous diploid plus a haploid, with a total effective genome size of 7.5 Gbp. We therefore estimate that our survey sequencing generated approximately 1.2% coverage.

**Figure 1:**
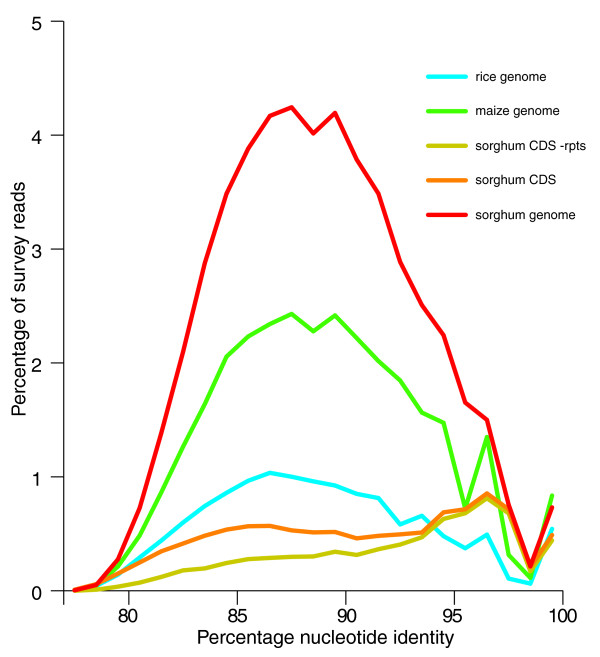
**Similarity of the *Miscanthus × giganteus *(*Mxg*) and other monocotyledon genomes**. A sequence survey of *Mxg *was compared to the sorghum whole-genome sequence (red line), the rice whole-genome sequence (blue line) and the maize whole-genome sequence (green line). In addition to the whole-genome sequences the survey was also compared to the predicted sorghum coding regions (CDS) unfiltered (orange line), and the sorghum coding regions with known transposon-related sequences removed (yellow line) using nucleotide BLAST. In all cases, the percentage nucleotide identity of the match (x-axis) is plotted against the percentage of the total reads from the survey with a given percentage identity to the relevant dataset (y-axis). No matches were observed with nucleotide identity below 75% at the e value cutoff used (10^-10^).

The unfiltered *Miscanthus *sequence reads were aligned to version sbi1 of the sorghum genome sequence [6] using nucleotide BLAST (BLASTN). A total of just over 51% of *Mxg *reads matched at e ≤ 10^-10 ^(Figure 1). BLASTN using the same parameters against all other GenBank DNA sequences, including whole genome shotgun data, showed that just 2,642 (0.7%) of reads match a sequence other than the sorghum nuclear genome while not matching the sorghum genome at this cutoff (including the 1,718 reads previously found to be highly similar to sorghum organellar genomes). This indicates that the *Mxg *genomic DNA template used for sequencing is unlikely to be contaminated by DNA from other organisms. The distribution of best-alignment lengths tracked the length distribution of the reads themselves, indicating that most alignments are full length. (The uptick at 100% identity seen in all curves of Figure 1 is largely attributable to a subset of BLAST alignments that are not full length.) The distribution of percent identity for *Mxg *reads aligned to the whole sorghum genome has a broad peak ranging from 85 to 92% (Figure 1). We then matched the *Mxg *survey reads to the predicted sorghum coding DNA sequence (genome annotation v. 1.4), again using BLASTN. In this case, a bimodal distribution is observed with a broad peak spanning 84 to 90% identity, and a sharp peak at 94 to 99% identity. Highly significant hits to both the sorghum whole genome and to the coding sequences were found to be evenly distributed across all ten sorghum chromosomes, indicating no obvious and substantial chromosomal loss in *Mxg *since divergence with sorghum.

We interpret the broad peak of reads with lower (84 to 90%) sequence identity as the result of highly repetitive coding sequences (for example, transposase-coding genes) within the sorghum coding region dataset. The sorghum gene models are known to contain retroelement-derived sequences despite repeat masking [6]. We therefore used the Plant Repeat Databases [30], which are largely composed of repetitive sequences from related grass genomes, including sorghum, to identify 1,322 likely repeat-derived coding regions within the sorghum coding sequence dataset using BLASTN at a 10^-6 ^e value cutoff. When these sequences were filtered from the coding region dataset, the matches with 80 to 90% identity substantially decreased, while the peak centered at 97% identity remained (Figure 1). The peak at lower levels of sequence identity is therefore likely to be the result of non-cognate matches of reads to repetitive coding regions such as the open reading frames of retrotransposons [31]. The remaining *Mxg *sequences matching at lower levels of sequence identity likely represent other high-copy protein-coding sequences not excluded by the filter. We consider the sequences present within the peak from 94 to 99% identity are likely non-repetitive protein coding sequences and conserved non-coding sequences. This level of similarity is consistent with the degree of nucleotide similarity observed in comparisons of the coding sequences for the C4 photosynthetic enzymes ribulose bisphosphate carboxylase/oxygenase (Rubisco), phosphoenolpyruvate carboxylase, and pyruvate orthophosphate dikinase between sorghum and *Mxg *[21]. A similar degree of sequence similarity is observed in comparisons of *Saccharum officinarum *ESTs to sorghum [6]. The *Mxg *survey sequences were also matched to the maize genome (release 3b.50) and the rice genome (release 6). The overall results were similar to those for the sorghum genome, except for significantly fewer matches overall (Figure 1). As expected from the phylogenetic proximity of sorghum to *Mxg *[9-11], sorghum is the most similar fully sequenced genome to *Mxg*. The peak at high percentage nucleotide identity is more obvious in maize, indicating that the conserved, coding DNA is substantially similar between maize and *Mxg*. Rice, which is not a member of the Andropogoneae, matches substantially fewer survey reads than maize.

The reads matching the sorghum predicted protein-coding genome at between 94% and 99% identity represent 3.4% of all total survey reads. We view this as a likely overestimate of the true proportion of protein coding DNA in the *Mxg *genome because some of these reads overlap exon-intron boundaries and contain noncoding as well as coding DNA. Thus, the percentage of reads matching exon sequences will be higher than the percentage of base pairs in the genome that are within exons. Such splicing boundary fragments are likely to be common because the average read length from the GS-FLX 454 sequences (229 bp) is comparable to the average exon sizes of approximately 250 bp observed for rice, maize and sorghum [6,30]. Assuming that full-length alignments of *Mxg *reads to the sorghum genome require at least 50 bp of overlap with sorghum exons that average 250 bp in length, then the *Mxg *exon space is estimated at 66% of the total sequence with significant matches to sorghum coding regions (Exon length/[Exon length + Read length - 2 × 50-bp miminum overlap] = 250/[250 + 229 -100]). Thus, our estimate of the single-copy conserved protein-coding DNA content is 2.2% of the *Mxg *genome. An estimated coding DNA content of 2.2% of the 7.5 Gbp *Mxg *genome would represent 165 Mbp. When divided by an average of 1,500 bp of coding sequence per gene, this leads to predictions of 110,000 genic loci per *Mxg *nuclear genome, or approximately 37,000 genes for each of the three component *Mxg *genomes. This number is comparable to recent estimates of gene number for sorghum and rice [6], but we note that our *Mxg *estimate would include any recent pseudogenes present in *Mxg *as these would not be distinguished by alignment of our short *Mxg *reads to sorghum, nor does it account for any gene loss that may have occurred after polyploidization.

### Repeat content of the *Mxg *genome

We used a number of techniques to characterize the repeat content of the *Mxg *genome. First, we compared the 454 survey sequences with the repeat classifications available from the Plant Repeat Databases [30] to classify each read on the basis of sequence similarity to known repeat sequences. Because the Plant Repeat dataset does not contain *Mxg *sequences but does include sorghum, we compared the abundances of hits within these data to a sample of 500,000 sorghum capillary sequence reads from the sorghum genome project, which unlike the assembled genome sequence are not depleted in unassembled (primarily centromeric, pericentromeric and rDNA) repetitive sequences, and thus more closely mimic our *Mxg *genome survey dataset. Because the *Mxg *and sorghum sequences were generated using different chemistry and the sorghum sequences were generated from plasmid templates propagated in *Escherichia coli*, the two datasets may have different genomic sampling biases. In addition, the read length of the capillary sequences is substantially longer. However, if the comparison provides similar results, it is both an indication of the similarity of repeat content of the two genomes and an assessment of the two sequencing chemistries in terms of their ability to represent complex plant genomes. Both read sets match many types of repeats, which is expected given the overall highly repetitive nature of Andropogoneae genomes. Thirteen percent of *Mxg *reads have a BLAST hit to the known repeat database at 10^-6 ^compared to 45% of sorghum reads. We attribute the large reduction in the proportion of *Mxg *matches to repeat sequences compared to sorghum to the shorter reads produced by the 454 technology and the absence of known *Mxg *repeats in the Plant Repeat Database. These results also indicate that a substantial number of *Mxg *sequences are not represented within known repetitive sequences.

**Figure 2:**
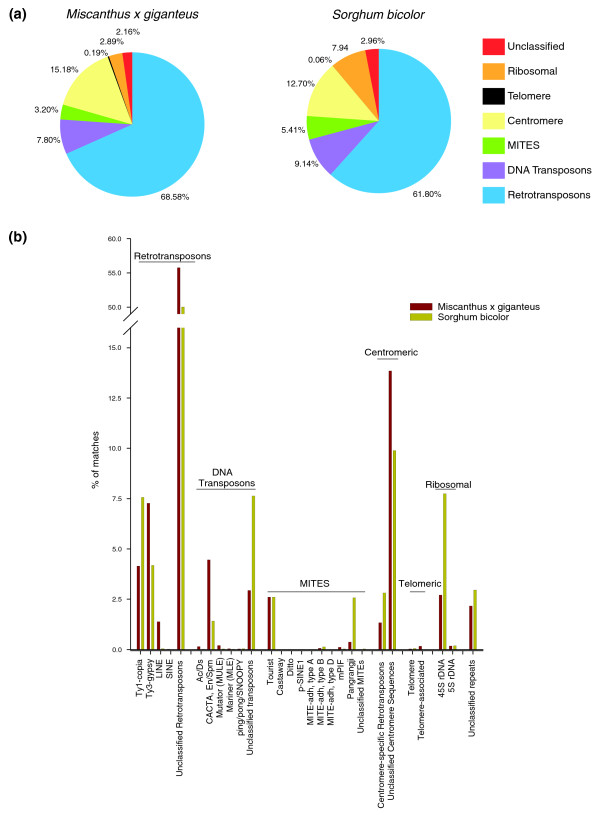
**Classification of repeats detected in *Miscanthus × giganteus *(*Mxg*) and sorghum by sequence comparison to the Plant Repeat Database**. Sequence surveys of *Mxg *and sorghum were matched to the Plant Repeat Database by nucleotide BLAST search. The proportion of repeats in each class for these two genomes was estimated by comparing the percentage of reads matching repeats of different classes in the database. **(a) **Proportion of repeats from surveys of the two species matching general classes of plant repetitive sequence. In both *Mxg *and sorghum, retrotransposons are the predominant class of repeats. Transposons are class II (DNA) transposons according to the designations in the Plant Repeat Database. **(b) **Further classification into repeat subfamilies, showing differing levels of miniature inverted repeat transposable element (MITE) and transposable element families in the two species. LINE, long interspersed nuclear element; SINE, short interspersed nuclear element.

The overall repeat composition of the two genomes based on the proportion of repeat classes is highly similar, with the retrotransposon class of repeats forming more than half of the best sequence matches in both cases (Figure 2). In both genomes the ribosomal repeats also occupy a significant fraction of the total, with the portion of the genome represented by rDNA in sorghum apparently substantially larger than that in *Mxg*. The rDNA of both species is likely to form a substantial portion of the total genome; however, the larger genome of *Mxg *may explain the observed lower percentage of repeats matching rDNA relative to sorghum. The genome of rice contains a 7 Mbp nucleolar organizing region, which constitutes 1.8% of the fully sequenced rice genome [32]. The nucleolar organizing region does not include the 5S ribosomal RNA region, and other dispersed ribosomal repeats. Thus, the estimates for the rRNA coding fraction of the genome in sorghum and rice are broadly consistent with the fraction of the rice genome responsible for rRNA production. The rDNA sequences obtained from the survey were fully consistent with the sequences previously deposited for the *Miscanthus *genus in GenBank, which further demonstrates the high purity of the *Mxg *genomic DNA template used for sequencing.

### Detection of new repetitive sequences in *Mxg*

We used the technique of non-cognate assembly [23] to identify novel high-copy-number repeats in the *Mxg *genome. As expected, a large number of *Mxg *reads assembled, indicating the presence of highly repetitive sequences. Based on a coverage estimate that our genome survey contains 1.2% of the *Mxg *genome, we calculate using the Lander-Waterman equations that three or more reads that assemble into one consensus sequence represent a multi-copy sequence present in the *Mxg *genome with a probability of 0.96 (thus, the null hypothesis is rejected at *P *< 0.05; see [23] for methods). A total of 20,774 'cluster' sequences were assembled from the *Mxg *survey reads that we predict are significantly repetitive (Additional file 3). These 'clusters' do not necessarily each represent an individual repeat family, since multiple clusters may be derived from different regions of a repetitive element. Also, all the repeats represented by these clusters are likely present in at least 100 highly similar copies per (total nuclear) genome in order to assemble; thus, this analysis will not detect many repeats of lower copy number. Sampling of the genome is a stochastic process; therefore, some repeats present in less than 100 copies may be detected, and some with more than 100 may not be detected. Of these assembled, repetitive sequences, 4,394 had no significant BLASTN match to the GenBank nt nucleotide database and 3,571 had no significant match to the draft sorghum genome, indicating, as with the comparison to the plant repeat database, that the *Mxg *genome harbors repetitive sequences not found in other sequenced plant genomes, including close relatives such as sorghum and maize.

Copy number of each assembled sequence was determined by computing the depth of coverage given by all genome survey sequences with 90% or greater similarity across their entire length. We found that 68% of all survey reads match the *de novo *detected repeats from the non-cognate assembly analysis. Note that in many cases, more than one repeat cluster will match a given read; in this case each read is counted only once. We thus estimate that a minimum of 68% of the *Mxg *genome is present in approximately 20,000 repeat families of 100 or more copies.

The ten most-abundant sequences in *Mxg *by this analysis are annotated in Table 1. The most abundant sequence in *Mxg*, a higher-order repeat related to the sorghum CEN38 centromeric repeat, has an estimated copy number of over 8,000 per genome in *Mxg *and over 2,000 in sorghum. This and similar (<90% identical) sequences are estimated to account for 0.45% of the *Mxg *genome and 1.02% of the much smaller sorghum genome (Table 1). The three-fold difference in nuclear chromosome number between the two species (2n = 20 for sorghum versus 3x = 57 for *Mxg*) likely accounts for the higher copy number of this centromeric sequence in *Mxg*. Since the CEN38 sequence is internally similar and forms higher-order repeat units, the table of most-abundant sequences based on 90% nucleotide similarity is headed by CEN38 family repeats, each of which share a basic repeat unit that is very similar (Additional file 3). The nine next most abundant sequences in *Mxg *after CEN38, which each are present in well over 10^3 ^copies per genome, are predicted to be retrotransposon-derived sequences, many of them also very similar to sequences associated with sorghum centromeres (Table 1).

**Table 1:** The ten most abundant repetitive sequence clusters detected in the *Miscanthus *genome

Cluster ID	Cluster length (bp)	Estimated copy number in *Miscanthus *genome	Copy number in sorghum genome	Best match in GenBank nt	Best match in sorghum genome	Best match in Plant Repeat Database
137362	3,866	8.82 × 10^3^	2.15 × 10^3^	AC169373 Sorghum	Chromosome 2	AF137608* Sorghum bicolor *centromere-associated repetitive DNA element CEN38
136340	1,441	1.97 × 10^4^	7.73 × 10^3^	No hits	Super contig 18	AY129008* Zea mays *CRM centromeric retrotranposon
137115	2,512	1.02 × 10^4^	1.90 × 10^3^	No hits	Super contig 20	AY828019* Oryza sativa *(*japonica *cultivar-group) clone CRR2_CH4-2 CRR2 retrotransposon
136416	1,855	1.32 × 10^4^	1.27 × 10^3^	No hits	Chromosome 10	AY129008* Zea mays *CRM centromeric retrotranposon
137059	2,586	8.27 × 10^3^	2.05 × 10^2^	No hits	Chromosome 2	ZRSiTERTOOT00157 giepum_438D03-1 giepum_Z438D03-1 Z438D03 retrotransposon
137200	3,655	5.61 × 10^3^	3.68 × 10^2^	No hits	Chromosome 9	SRSiTERTOOT00084 leviathan_98N8-1 leviathan_98N8-1 retrotransposon
137015	2,882	6.83 × 10^3^	7.5 × 10^1^	No hits	Chromosome 7	AF078902.1* Sorghum bicolor *centromere element pHind12
137326	3,527	5.56 × 10^3^	3.30 × 10^2^	AF11417 Sorghum	Chromosome 2	AZ922428 MRCot4C10 *Sorghum bicolor *MRCot *Sorghum bicolor *genomic similar to *Sorghum bicolor *centromere element pHind12
137201	3,647	5.28 × 10^3^	2.16 × 10^2^	No hits	Chromosome 10	ZRSiTERTOOT00156 giepum_333J11-1hdnm giepum_Z333J11-1hdnm Z333J11 retrotransposon
135786	2,456	7.82 × 10^3^	1.89 × 10^2^	No hits	Chromosome 4	ORSiTERTOOT00275 osr10 retrotransposon

**Figure 3:**
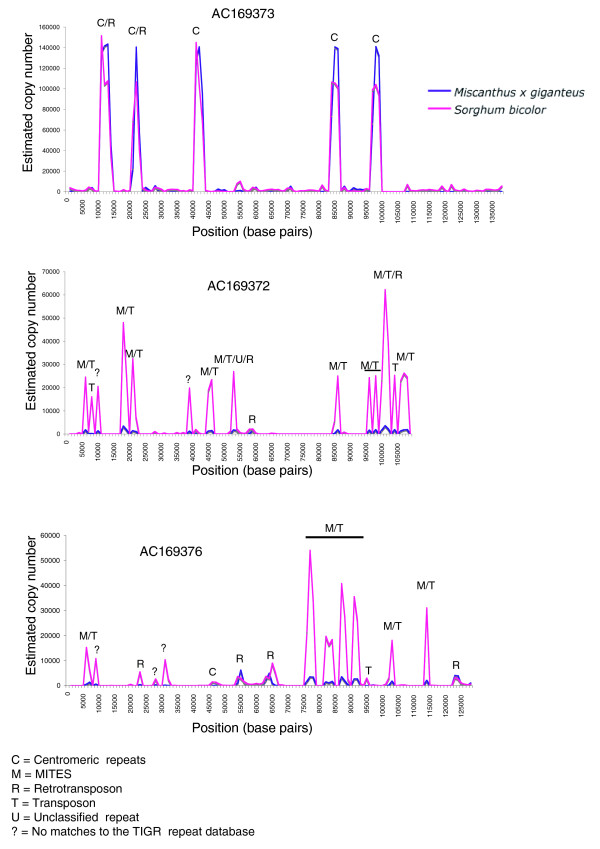
**An estimation of copy number of sequences present in three sorghum genomic sequences in sorghum and *Miscanthus × giganteus *(*Mxg*)**. Copy number was estimated for regions of the sorghum genome in both sorghum and *Mxg*. Shown are three completed sorghum BAC sequences, one centromeric and two euchromatic. Sorghum copy number was estimated by matching to a sequence dataset of whole-genome sorghum shotgun sequences (red) and the *Mxg *copy number estimated by comparison to the 454 survey reads (blue) using a blastZ alignment within a 1,000-bp sliding window. The estimated genomic copy number based on the number of reads matching each window (y-axis) is plotted against the position of the window on the BAC (x-axis). The nucleotide identity cutoff for this analysis was 90%. The regions of greatest copy number on BACs AC169372 and AC169376 predominantly match miniature inverted repeat transposable elements (MITEs), transposons and retrotransposons, which are significantly more abundant in sorghum, while AC169373 contains highly abundant centromeric repeats, for which the *Mxg *and sorghum copy numbers agree closely.

We also compared the relative abundance of specific homologous repetitive sequences in sorghum with the abundance of highly similar high-copy sequences in *Mxg*. This was accomplished using a strategy of alignment of sorghum bacterial artificial chromosome (BAC) sequences to highly similar reads from the *Mxg *survey and the 500,000 sorghum capillary trace sequences, and using this alignment to calculate the approximate copy number of similar sequences in each genome [23]. This method allows the comparison of overall repeat class composition (Figure 2) with the estimates for genomic copy number of specific homologous repeat families that are present in both species (Figure 3).

Three sorghum BAC sequences were investigated from the Joint Genome Institute BAC dataset, one likely to derive from a centromeric or pericentromeric region (accession number AC169373) and the others (accession numbers AC169376 and AC169372) likely representing euchromatic regions. The euchromatic BACs show that the repetitive sequences present in these regions of the sorghum genome are almost all higher copy number in sorghum than *Mxg*. This is particularly notable for miniature inverted repeat transposable element (MITE) sequences. MITEs are known to undergo rapid increases in copy number [33] and this process likely has occurred for the sorghum high-copy MITEs since ancestral divergence with *Mxg*. Notably, the centromeric repeats from the pericentromeric BAC have the same or higher copy number in *Mxg *as in sorghum. This observation possibly reflects more conserved sequence in centromeric repeats than the repetitive euchromatin since the divergence of *Mxg *and sorghum. The slightly higher abundance of the repeat sequences is consistent with the significantly higher chromosome number of *Mxg *[18,34] but could also be the result of differential selection by the two sequencing chemistries employed in this comparison (Sanger for sorghum, 454 for *Mxg*) [23]. The strong correlation between the sorghum and *Mxg *repeat content for AC169373 indicates that any such bias is not severe.

### Much of the sRNA transcriptome of *Mxg *corresponds to repeat sequences identified in the genome survey

**Figure 4:**
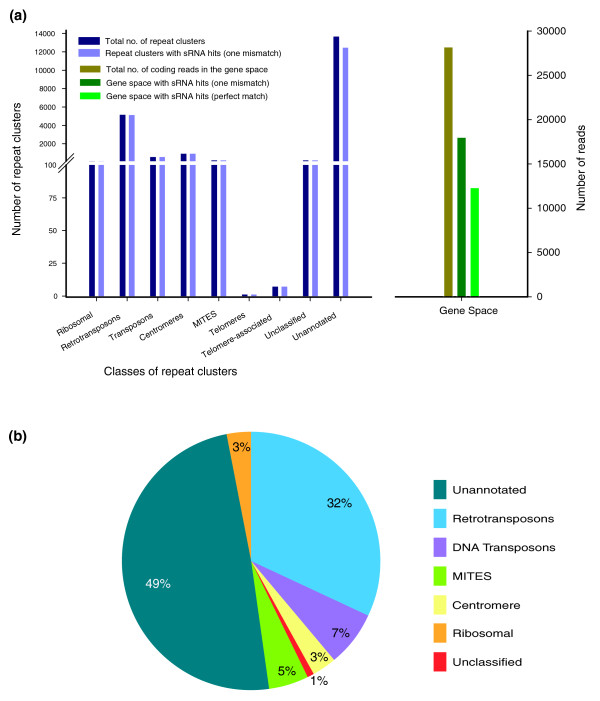
**Much of the small RNA transcriptome of *Miscanthus × giganteus *(*Mxg*) matches high copy number genomic repeats**. **(a) **Number of *Mxg *repeats, or gene space sequence reads, matching a small RNA (sRNA), as determined by matching repeat sequences to sRNA signatures produced by sequencing sRNA from three *Mxg *tissues. Repeats are annotated by broad category where known. Unclassified repeats match a sequence in the database without an assigned category; unannotated repeats do not have a database match. Gene space reads are genome survey reads that match sorghum filtered coding sequences (Figure 1). **(b) **Percentages of small RNA produced by different repeat classes. Normalized abundance of small RNA signatures was calculated in transcript per quarter (TPQ) million reads. In addition to the data shown, telomeres and telomere-associated repeats together produced 0.09% of the total amount of sRNA (a percentage too small to effectively display in the chart).

To correlate the repeat sequences identified in this study with the sRNA transcriptome of *Mxg*, we compared the repeat consensus sequences of clusters constructed using non-cognate assembly with raw data from deep sequencing of sRNAs from *Mxg *rhizomes, stems and inflorescences [35,36]. By sequence matching of the sRNA sequences to our identified repeats, we found that the vast majority (>94%) of the identified genomic repeat sequences also appear to match measurable amounts of sRNAs, judging by the presence of matching signatures (Figure 4a). This is the case regardless of the class of repeat since almost all repeat families (including rDNA, transposons, retrotransposons and most repeats of unknown classification) shown in Figure 4a match at least one sRNA signature. In order to compare this result with likely single-copy sequence from the protein-coding gene space of *Mxg*, we also compared the sRNAs to the 'gene space' survey reads. These are the survey reads that matched the sorghum gene models after filtering for transposon sequences (Figure 1). With one mismatch allowed in either case, a total of 586,765 sRNA signatures matched the repeat clusters, while 194,821 matched the gene space survey sequences. With no mismatches permitted, 64,339 reads matched the gene space survey sequences. Whether a single mismatch was allowed or not, a relatively small proportion of gene-space reads match a sRNA signature (Figure 4a). Thus, the sRNA profile and the genome survey combine to produce strong evidence of abundant repeats in *Mxg *that are the origin of much of the cellular sRNA component.

Most sRNAs from cereals, including grasses, are in the 24-nucleotide size range [35] and thus are likely siRNAs. In the three sRNA libraries sequenced and analyzed for this study, between 59 and 70% of the sRNAs were 24 nucleotides in size, and 23 to 30% of the sRNAs from these libraries matched the repeat sequences identified from the genome survey. As shown in Figure 4, retrotransposons (class I transposons) produced the largest portion of sRNA (32%) followed by DNA transposons (class II transposons). Given the high abundance of retrotransposons in the genome, these results suggest that siRNAs derived from repeats are the major component of the *Mxg *small-RNA transcriptome. Similar findings were reported in rice and *Arabidopsis *[26,36].

### Correlation between number of sRNA matches and repeat copy number

**Figure 5:**
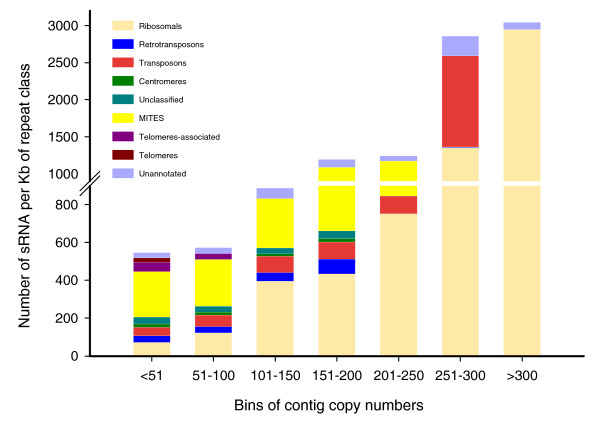
**Correlation between repeat copy number and amount of small RNA per kilobase of matching repeat sequences**. Repeats were binned according to their estimated copy number in the *Mxg *genome and then divided into categories as in Figure 4a. The number of small RNA signatures matching the repeat sequence in each category and copy number class divided by the estimated total genomic size of the repeat class in kilobases is plotted on the y-axis. MITE, miniature inverted repeat transposable element.

Since a whole-genome sequence is not available for *Mxg*, the copy number and number of kilobases of each repeat present in the genome were estimated. This was calculated from the sequence coverage of each repeat with respect to the expected coverage and the length of the repeat sequence. Copy number and repeat size estimates allowed us to normalize the number of sRNA matches to the expected content of each repeat in the genome. We observed a strong correlation between the estimated copy number of a repeat in the genome and the total amount of 24-nucleotide sRNAs matching the sequence of the repeat in the combined data from the three libraries (Figure 5). This correlation was observed even though the number of sRNA matches was normalized to the estimated total genomic content of the repeat. The relationship between sRNA number and minimum copy number of the respective bin is approximately linear (R^2 ^= 0.84). The sRNA number from each bin differs from the even distribution expected under the null hypothesis in a statistically significant manner (*P *< 0.001 using the chi square test). We repeated this analysis using different bin sizes (from 25 to 200), and in each case the relationship was confirmed.

Although retrotransposons constitute the largest proportion of the genome of any repeat (Figure 2) and produce the largest portion of sRNAs (Figure 4b) in the transcriptome, they have a relatively small amount of sRNA matching per kilobase of sequence. The single highest copy-number repeat, the rRNA, maintains the highest rate of sRNA matching per kilobase of repeat sequence in the genome (Figure 5), although a substantial proportion of these sRNAs may be produced as a result of ribosome degradation rather than sRNA biosynthesis. In the same context rRNA was previously reported as a major source of sRNAs in *Arabidopsis *[26]. The less abundant MITEs matched sRNA at a higher rate than retrotransposons and a lesser rate than rRNA (Figure 5), providing evidence for active sRNA biogenesis derived from MITEs in the *Mxg *genome. An exception to the correlation between copy number of a repeat and sRNA production is that few sRNAs matched retrotransposons of copy numbers higher than 200 (Figure 5). The highest copy-number retrotransposons, unlike MITEs, may therefore be relatively inactive in terms of sRNA biogenesis. Thus, small RNA production may reflect the degree of heterochromatic silencing of repeats in addition to copy number.

### High-coverage survey sequencing reveals ancestral rDNA

The substantial sample of the RNA-coding genome produced by the *Mxg *genome survey presents an opportunity to test the hypothesis that *Mxg *originated from a wide cross between two parental species [11], producing a highly productive but sterile hybrid. Variants within rDNA repeats can be identified by mining shotgun genome sequence data for variant units [37]. We used the same approach to mine reads spanning an informative region of the internal transcribed spacer (ITS) [38] from the 454 genome survey of *Mxg*. Reads matching rDNA were filtered in order to remove those containing low quality base pairs that could cause false phylogenetic results. Reads producing an orthologous aligned block from the second intergenic spacer (ITS2) [39] were then aligned with known rDNA sequences. Since it has been proposed that the parents of *Mxg *are *M. sinensis *and *M. sacchariflorus *[11], sequences from these species were included in the alignment in addition to the existing *Mxg *rDNA sequences (accession numbers AJ426562 and AJ426563) [9] and other closely related Andropogoneae rDNA sequences. A phylogenetic tree was then generated from the ITS2 block using the Bayesian inference method [40,41]. Thus, the 454 survey of the *Mxg *genome was used to sample the population of rDNA, and to explore the parentage of this complex polyploid.

**Figure 6:**
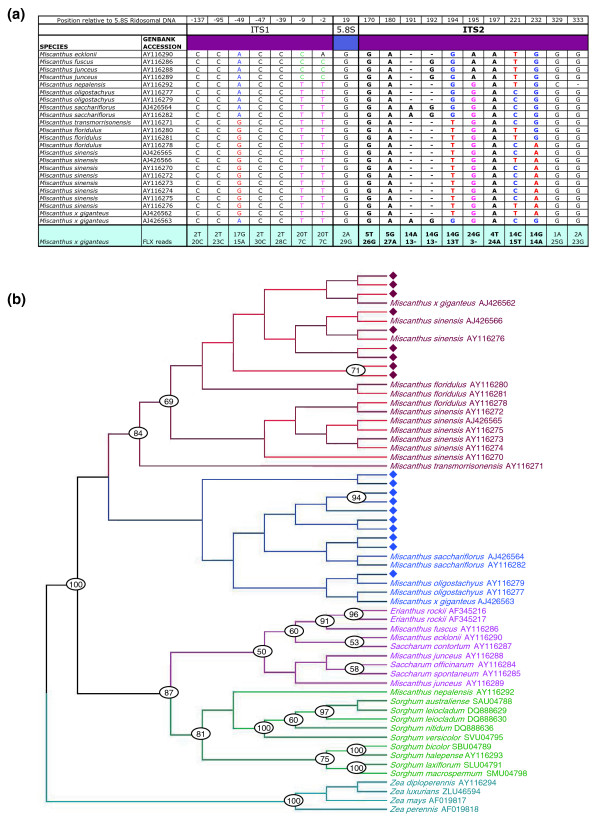
**Phylogenetic analysis of *Miscanthus × giganteus *(*Mxg*) based on nuclear ribosomal DNA**. **(a) **Sites of variation in a nucleotide alignment of the ITS1, 5.8S and the ITS2 regions of the rDNA from various *Miscanthus *species and *Mxg *survey reads. Reads from the *Mxg *genome survey that matched the internal transcribed spacers (ITS1 and ITS2) and the 5.8S rRNA were manually aligned using Sequencher and McClade to *Miscanthus*, Maize, sorghum and *Saccharum *sequences from GenBank and variable residues identified. **(b) **Phylogenetic tree of *Mxg *survey reads together with related species. A Bayesian phylogenetic analysis of the residues from a 150-bp region of ITS2 spanned by several complete reads from *Mxg *and shown in bold in (a) was performed using the general time reversible (GTR) model of substitution and a gamma distribution of the rates of substitutions. Parameters estimated from the last 5,000 trees were used to calculate a posterior probability at each node and draw a 50% majority rule consensus tree (b). The numbers at the nodes indicate the percentage confidence in the branches as assessed using the posterior probabilities. The diamonds represent the individual *Mxg *survey reads from this region.

The results of this analysis are shown in Figure 6. It can be seen that the rRNA species in the *Mxg *genome match either *M. sacchariflorus *(ten reads) or *M. sinensis *(nine reads). This result is consistent with the proposal that *Mxg *descended by asexual propagation from the progeny of a hybridization event between *M. sacchariflorus *and *M. sinensis *parents.

## Discussion

We have completed a survey of the *Mxg *genome sufficient to give a snapshot of its overall composition and to aid planning for genome sequencing projects aimed at the large genome of *Mxg *and other complex, polyploid crops. The 454 technology used to sequence approximately 1.2% of the *Mxg *genome produced a comparable repeat profile to Sanger capillary shotgun data for sorghum, indicating that the two technologies exhibit a similar level of sampling bias and that much of the repeat content is indeed shared by the genomes of these species. Our results also indicate that GS-FLX 454 pyrosequencing is capable of producing similar datasets in terms of genome sampling to that used to successfully assemble sorghum. However, assembly of the sorghum genome was achieved using very different data: capillary sequence data with coverage of 7× to 8×, a much longer read length than that provided by the GS-FLX method, and reads in paired-end configuration from relatively large molecules. Therefore, we have demonstrated the genome sampling delivered by the 454 method is suitable for whole-genome survey work. However, greater coverage, further advances in read length and paired-end technology may be necessary before true *de novo *whole-genome sequencing of a genome such as *Mxg *is feasible using non-Sanger sequencing [42].

The combination of sRNA profiling with the genome survey extends to the grasses the earlier findings from *Arabidopsis *that high-copy repeats are likely to produce large amounts of siRNA [26]. In addition, this combination allowed us to determine a clear correlation between copy number of genomic repeats derived from a survey, and sRNAs matching a repeat, even when the total amount of sequence of the repeat class present in the genome is accounted for. Therefore, it may help to give insights into the sRNA production process (since sRNA levels appear in most cases to rise in proportion to both copy number and content of a sequence in the genome). A survey of sRNA adds significantly to a genomic sequence survey alone, since it provides confirmation that the repeats detected in the survey are both present and biologically active. Many of the survey reads that matched sorghum predicted coding regions also matched sRNA signatures, although the percentage was not as high as for repeats. While the known retrotransposon sequences were removed, it may be possible that repetitive sequences remain within the set of 'gene space' reads. Full characterization of the *Mxg *gene space is needed to fully interpret this result.

The abundance of sRNA is, when averaged over all the repeats in a copy number class, roughly in linear proportion to the content of the repeat in the genome. This is true even once the result is normalized for the number of kilobases of the repeat present in the genome, as in Figure 5. This result is consistent with previous surveys [26,36]; however, to our knowledge it has not yet been shown that repeat copy number alone, regardless of the amount of DNA for the repeat present in the genome, is a strong indicator of likely sRNA abundance. While there are many repeats that form exceptions to this relationship, it holds true for the average of repeats in a copy number class across a range of bin sizes. The underlying biology of this relationship in plant genomes warrants further investigation.

The sRNA survey may also give insight into which classes of transposable element are the most biologically active in terms of sRNAs at the time of the survey. The MITEs present at relatively low copy number in the genome survey data produce a significant fraction of the sRNA, indicating that MITE activity is actively silenced in the vegetatively propagated *Mxg *[43]. The correlation between sRNA production and actual transposition activity is currently unknown. Thus, the sRNA could indicate either actively transposing elements or strong and effective silencing. Activation of an abundant class of transposons, however, has the potential to significantly impact *Mxg *genome function by generating somaclonal variants that, even if deleterious, may be maintained within *Mxg *populations because of triploid sterility and exclusively vegetative propagation. While the effect of mutagenic transposon activity is likely to be moderated by the redundancy provided by the triploid genome of *Mxg*, it is possible that observed phenotypic variation among *Mxg *accessions could derive from transposon activity (a concern in a potential fuel crop). Thus, further research is necessary to determine whether the sRNA signatures observed are indicative of active transposition of class I or II transposons.

We demonstrate that *Mxg *contains rDNA similar or identical to that of both presumed parents, *M. sinensis *and *M. sacchariflorus*, in approximately equal quantities. Nuclei in *Mxg *with both one and two nucleoli have been observed [17] with the two being less frequent. Our result is compatible with the survival in *Mxg *of two distinct rDNA species, which may represent the two nucleoli previously observed [17] in some cells. Ribosomal RNA genes are propagated in a manner that tends to unify all rDNA units within a hybrid species after reproductive isolation in a process known as concerted evolution [37]. While an organism such as *Mxg *that propagates through clonal divison may not be expected to undergo rapid homogenization of rDNA units, evidence from parthenogenetic lizard species suggests that concerted evolution can operate rapidly even within asexually reproducing hybrids [44]. Although we are not aware of any evidence from plants for rapid homogenization of rDNA during asexual propagation, our analysis of rDNA supports the view that *Mxg *is derived by asexual propagation of a hybridization event sufficiently recent to have not yet resulted in a complete homogenization of rDNA. Further research is necessary to identify the likely rate of homogenization, which could yield a maximum estimate for the age of this asexually propagated hybrid.

*Mxg *is sterile, and self-incompatibility is common within the *Miscanthus *genus. Although a low-resolution genetic map has been developed for *M. sinensis *[45], these resources are not yet sufficient for positional genetics. Thus, the primary use of *Mxg *sequence information will be to enable functional genomics efforts. These goals could be achieved with detailed knowledge of the protein-coding gene space sequence, which, in turn, could be accomplished using 454 pyrosequencing. Despite its (currently) shorter read lengths than capillary technology, 454 sequencing will likely be suitable for gene-space sequencing, since the assembly would only need to produce contigs in the range of 10 kbp, and the repeat sequences shown to be highly abundant here could be removed or reduced prior to sequencing. Purification of gene-space could be accomplished by direct 454 sequencing of mRNA [46] or by enrichment of the protein-coding fraction of the genomic DNA via methods such as methylation filtration or C_0_t filtration [47]. Since mRNAs vary widely in abundance and are not expressed in all tissues, it is difficult to arrive at a whole-gene-space sequence using mRNA alone, even with the effective normalization procedures now available. Detailed mRNA sequence is often, however, necessary to interpret genomic DNA sequence even in genic regions and thus makes a useful addition to gene space sequence. Thus, efforts that target the protein-coding fraction of the genome, using gene-space enrichment combined with mRNA sequencing, may represent the most promising approach in the immediate future. Such an approach has already been demonstrated for maize and sorghum [48,49] using genomic fragments cloned into *E. coli *strains that restrict methylated (hence typically repetitive) inserts.

## Conclusions

We have shown that the 454 survey and non-cognate assembly method is capable of sampling genomic repeats in detail from an uncharacterized genome. We show that by combining the resultant repeat catalog with sRNA sequencing, a detailed knowledge of both the repetitive parts of the genome and the sRNA derived from them can be obtained rapidly. The survey also gives access to rDNA sequence in a way that makes data available on the parentage of complex crosses such as *Mxg*. Such data are important because it may be feasible to recreate and improve upon *Mxg *as a biofuel crop if the fertile parents of the original hybridization event can be identified. The survey data provide sequences of several highly variable genomic regions, in addition to the rDNA sequence, that can be utilized to identify the most likely parental genotypes of *M. sacchariflorus *and *M. sinensis*, information that could be used to recreate *Mxg*. The high level of nucleotide similarity of the *Mxg *coding regions to sorghum demonstrated here promises that the sorghum genome may provide a useful template for an assembly-to-reference strategy for the *Mxg *gene space. Improvements in costs and efficiency offered by 454 and Illumina sequencing make obtaining gene space sequences of *Miscanthus *and other Andropogoneae species with similarly complex genomes using short read technologies a feasible goal.

## Materials and methods

### Plant material

The *M. × giganteus *accession 'UIUC' was obtained from a stand of *Mxg *originally established in the early 1990s on the Ornamental Horticultural Research Farm at the University of Illinois at Urbana-Champaign, using rhizomes obtained from the Chicago Botanical Garden. Rhizomes from this clonal stand were propagated in potted soil in a greenhouse under controlled conditions of 14 hour days and 10 hour nights at 22 to 25°C, and a brief watering each morning. Leaves from newly initiated shoots were sampled for DNA analysis.

Each flow cytometry data point shown was conducted on nuclei isolated from young leaf tissue from a single greenhouse-grown plant of the genotypes described, obtained from a collection at the University of Illinois maintained by Dr J. Juvik. Nuclei stained with propidium iodide were measured for DNA content against chicken red blood cell standards as described by Arumuganathan and Earle [50]. The analysis was done at the Flow Cytometry and Imaging Core laboratory, Benaroya Research Institute at Virginia Mason, Seattle, Washington.

### *M. × giganteus *nuclear DNA isolation and sequencing

Nuclei were isolated from 20 g of young *Mxg *leaf tissue using a protocol modified from Swaminathan [23]. Instead of using a sodium lauryl sulfate and protease K to lyse the nuclei, they were lysed by an incubation at 60°C in CTAB lysis buffer (2% CTAB,100 mM Tris pH9.5, 1.4 M NaCl, 1% PEG 6000, 20 mM EDTA and 0.25% β-mercaptoethanol). The lysate was extracted twice with neutral, tris buffered, 24:23:1 phenol:chloroform:isoamyl alcohol followed by an extraction with chloroform:isoamyl alcohol (23:1). Nuclear DNA was precipitated using isopropanol. The spool of genomic DNA was washed with 70% ethanol, dried and resuspended in TE. Any contaminating RNA was removed by an incubation at 37°C with RNaseA and repurification through additional phenol:chloroform and chloroform extractions, followed by ethanol precipitation and resuspension in nuclease free water. *Mxg *nuclear DNA (5 μg) was supplied to the Roy J Carver Biotechnology Center at the University of Illinois at Urbana Champaign [51] for library preparation and DNA sequencing using the Roche Genome sequencer FLX system (GS-FLX).

### Datasets

Sorghum genome sequences were obtained from two sources. To characterize repeat content and copy number, we downloaded 500,000 whole genome shotgun sequences, the sorghum_bicolor.022 portion of the sorghum whole genome shotgun dataset available from Trace Archive. These reads are unfiltered for repeats and hence comparable to the data generated by the FLX runs.

Comparisons to sorghum used the current versions of the genome assembly and coding sequence annotation: sbi1 (genome sequence) and Sbi1-4 (coding sequences). The BAC sequences for the copy number analysis were downloaded from [GenBank: AC169372, AC169373 and AC169376]. For classification of known repetitive sequences we used the plant repeat database [30]. To match *Mxg *reads to the sorghum draft genome and derived sequences, BLASTN was used at expect value cutoff 10^-10 ^and with the repeat filter on and other settings as default. For comparison between survey sequences and the Plant Repeat Database, BLASTN was used at expect value cutoff 10^-6 ^and with repeat filter off. Matches to each class of repeat were counted and percentage of each repeat class in the genome calculated, using the codes incorporated into the IDs of the plant repetitive sequences in the Plant Repeat Database [30].

### Assemblies to detect novel repeats in *Mxg*

Overlapping, non-cognate short reads generated by the GS-FLX run were assembled using the 64 bit manyreads build of phrap ver 1.080721 [52]. All assemblies were done on a 16-core Intel Xeon 2.93 GHz server with 128GB DDR2 RAM. Parameters for the phrap assembly were the same as those used in [23], except that the minimum overlap length for two reads to be assembled was 14 bases at 100% nucleotide identity.

### Copy number estimation

The 366,648 *Mxg *reads from the GS-FLX run and 500,000 sequences from sorghum whole-genome shotgun project were first matched using BLAT [53] at 90% identity cutoff to three fully sequenced sorghum BACs (accession numbers AC169372, AC169373 and AC169376). The *Mxg *and sorghum sequences were also matched in the same way to the repeat clusters produced by the non-cognate assembly. BLAT was run with default parameters of a tile size of 11 and a minimum score of 30. Fully cognate alignments of the sequences identified using BLAT as matching the BACs or repeat clusters were then created using blastz [54]. Blastz was run with default parameters, aligning reads to both strands with a word size of 8, no chaining and a gap-extension penalty of 30. The threshold for maximal scoring segment pair (MSP) and gapped alignment threshold were set to 3,000. Any sequence that did not match these alignment criteria was excluded from the copy number analysis. The number of sequences that aligned to the sorghum BAC window or *Mxg *repeat cluster was calculated from the blastz output and used to calculate copy number using the equation:(1)

N is the number of sequences that match a window size of w, L is the average length of the sequence and c the estimated coverage of the survey. For *Mxg*, we used the estimate of 0.012 (1.2%) coverage of the total genome. Price *et al*. [55] estimated the haploid genome size of sorghum to be 818 Mbp (note that the draft assembly excludes telomeric, centromeric and rDNA repeats). We thus calculated the sorghum genome coverage as coverage of the haploid genome for the 500,000 sorghum capillary sequences at 0.63 (63%). Repeats were sorted according to the estimated percentage of each in the *Mxg *genome, calculated from the repeat cluster sequence length, genome sequence length and estimated copy number. The ten most abundant repeats were identified by excluding all but one cluster annotated with the same top hit to the Plant Repeat Database.

### sRNA isolation, sequencing and analysis

A total of 6,736,105 sRNA signatures (18 to 32 nucleotides in length) from three Solexa flow cell lane data sets produced from three libraries from greenhouse-grown *Mxg *'UIUC' tissues (leaf, inflorescence, and rhizome) were mapped to 20,774 *Mxg *repeat clusters (derived by non-cognate assembly of genomic survey reads, above) using GMAP (Genomic Mapping and Alignment Program) [56]. The program parameters were set to report all possible hits for every sRNA signature. Only sRNA hits with coverage equal to 100% and matching identity greater than or equal to 94% were used in the subsequent analyses. These parameters allow only one mismatch between the sRNA signature and the aligned genomic locus. For the gene space survey reads, this analysis was also performed with 100% coverage required and also 100% identity.

For the analysis of sRNA matches versus copy number, sRNA signatures were matched to the repeat cluster collection using GMAP as above, with up to one mismatch per signature allowed. For each repeat sequence, the number of small RNAs matching the sequence was divided by the estimated number of kilobases of the repeat present in the *Mxg *genome. This estimate of kilobases per genome was arrived at by using Equation 1 to give an estimate of copy number multiplied by the length of the sequence in kilobases. The total number of sRNA signatures per kilobase in the genome for the repeat clusters was then summed across each class of repeat within copy number bins; for example, all sRNA matching all MITE sequences expected to occur between 151 and 200 times per genome was summed to create a single value for MITE sRNA matches in this repeat class.

### Phylogenetic analysis

Reads that matched *Mxg *nuclear ribosomal sequences [GenBank: AJ426562 and AJ426563] at 90% identity were identified using BLAT. There were 114 unique reads that matched either one or both *Mxg *nuclear ribosomal sequences using default BLAT parameters. Additional *Miscanthus*, *Zea*, *Saccharum *and *Sorghum *nuclear ribosomal sequences previously used to construct phylogenetic trees for *Miscanthus *and *Saccharum *[9] were obtained from GenBank. The sequences and the reads with a minimum overlap of 50 bp and 80% identity to AJ426562 were assembled using Sequencher (Gene Codes, Ann Arbor, MI, USA). Regions of variations within the reads were quantified. The greatest variation lay within a 150-bp region of ITS2 (Figure 4). The alignment matrices were then trimmed by removing gaps present in more than 95% of the sequences and truncating the sequence limits to this 150-bp region, to account for poor resolution of indels, especially in homopolymeric regions, in the sequencing method, and maximize the number of survey reads used in the analysis. A total of 19 *Mxg *survey reads met these criteria throughout the 150-bp ITS region and were retained for this analysis. Phylogenetic relationships were estimated using Bayesian inferences conducted in MrBayes version 3.1.2. The general time reversible model taking into account the shape of gamma distribution was used, as suggested by Modeltest3.7, as the most appropriate model of sequence evolution. The analysis was run for 1.5 × 10^6 ^generations with sampling at intervals of 100 generations, thus generating 15,000 sampled trees. A 50% majority rule consensus tree was generated to assign a posterior probability for each node using the last 5,000 trees sampled. The tree was visualized in Dendroscope [57].

### Data access

The 454 sequence data described in this publication have been deposited in the NCBI Sequence Read Archive [SRA:SRA010791.2]. The sRNA data discussed in this publication have been deposited in NCBI's Gene Expression Omnibus [GEO:GSE20056].

## Abbreviations

BAC: bacterial artificial chromosome; bp: base pair; EST: expressed sequence tag; ITS: internal transcribed spacer; MITE: miniature inverted repeat transposable element; *Mxg*: *Miscanthus × giganteus*; rDNA: DNA encoding the tandem array of rRNA genes; siRNA: small interfering RNA; sRNA: small RNA.

## Competing interests

The authors declare that they have no competing interests.

## Authors' contributions

KS participated in the design, carried out all DNA-related experiments, performed most of the data analysis, prepared Figures 2, 3 and 6 and helped draft the manuscript. MA performed the sRNA data analysis, prepared Figures 4 and 5 and helped with the manuscript. KV wrote scripts, performed non-cognate assembly, copy number estimation and annotation of the repeat survey data. EDP performed the RNA purification from *Mxg *tissues, coordinated sequencing-by-synthesis of sRNAs, processed the raw sequencing data and helped with the manuscript. IH helped develop the comparative approaches with the sorghum genome. DR participated in the design, helped with coverage calculations, proofed the manuscript and helped develop comparative approaches to sorghum. AKA performed flow cytometry and interpreted genome size measurements. RM directed genome size measurements and helped with comparative genomics discussions. PJG and BCM directed sRNA sequencing and contributed to the manuscript. SPM participated in the design, grew *Mxg *tissue for DNA and sRNA extraction and helped with the analysis and manuscript. MEH conceived the study, wrote scripts, performed some analyses, prepared Figure 1 and drafted the manuscript. All the authors read and approved the manuscript.

## Supplementary Material

Additional file 1 Estimates of nuclear DNA content in several Miscanthus accessions, by flow cytometry. Click here for file

Additional file 2 Flow cytometric histogram of *M. × giganteus *nuclei stained with propidium iodide. Click here for file

Additional file 3 A table (tab delimited text format) of all repetitive sequences detected in the *Miscanthus *genome. Click here for file
