# Supplementary material for: Genomic and small RNA sequencing of Miscanthus × giganteus shows the utility of sorghum as a reference genome sequence for Andropogoneae grasses
Source: Genome Biol. 2010 Feb 3;11(2):R12. doi: 10.1186/gb-2010-11-2-r12 (PMC2872872; doi:10.1186/gb-2010-11-2-r12)
Supplement: Additional file 1 — Estimates of nuclear DNA content in several Miscanthus accessions, by flow cytometry. [file gb-2010-11-2-r12-S1.pdf]

Sample name	Sample ID	Sample<br>G0+G1 mean	Standard (int.)<br>G0+G1 mean	DNA content<br>(pg/2 or 3C)	St. Dev<br>±
Miscanthus x giganteus	***#1***	596.44	189.16	7.88	
UIUC		655.38	206.20	7.95	
		719.43	227.62	7.90	
		757.07	249.11	7.60	
				**7.83**	**0.158**
			Mbp/1C	***3837.69***	***77.62***
Miscanthus sinensis	***#3***	549.69	185.04	7.43	
Autumn Light		605.02	202.17	7.48	
		664.57	216.53	7.67	
		730.25	236.90	7.71	
				**7.57**	**0.138**
			Mbp/1C	***3837.69***	***77.62***
Miscanthus sinensis	***#4***	448.87	198.20	5.66	
Silberfeder		494.05	215.74	5.73	
		539.15	239.17	5.64	
		591.37	265.38	5.57	
				**5.65**	**0.064**
			Mbp/1C	***3837.69***	***77.62***
Miscanthus sacchariflorus	***#5***	415.82	186.92	5.56	
Amur Silver Grass		459.96	204.18	5.63	
		505.01	216.65	5.83	
		555.26	234.46	5.92	
				**5.74**	**0.167**
			Mbp/1C	***3837.69***	***77.62***
Miscanthus sacchariflorus	***#6***	364.11	201.34	4.52	
Blue Stem Nursery		399.98	221.10	4.52	
		437.21	246.29	4.44	
		479.04	272.92	4.39	
				**4.47**	**0.066**
			Mbp/1C	***3837.69***	***77.62***

1 pg= 980 mbp
