# Supplementary material for: Genomic and small RNA sequencing of Miscanthus × giganteus shows the utility of sorghum as a reference genome sequence for Andropogoneae grasses
Source: Genome Biol. 2010 Feb 3;11(2):R12. doi: 10.1186/gb-2010-11-2-r12 (PMC2872872; doi:10.1186/gb-2010-11-2-r12)
Supplement: Additional file 2 — Flow cytometric histogram of M. × giganteus nuclei stained with propidium iodide. [file gb-2010-11-2-r12-S2.pdf]

Supplemental Figure 1

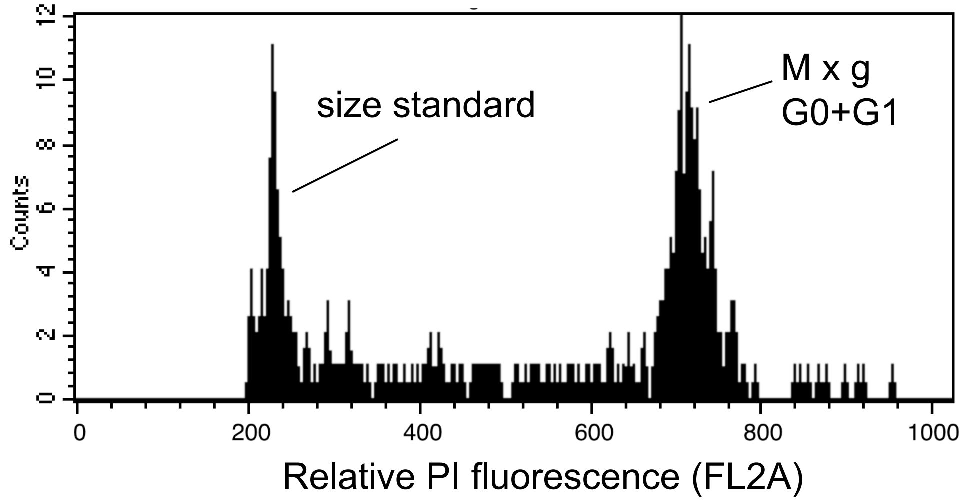

Supplemental Figure 1: Flow cytometric histogram of M. x giganteus nuclei stained with Propidium Iodide
